# Molecular Characterization of Clarithromycin Resistance in *Helicobacter pylori* Strains

**DOI:** 10.5152/tjg.2023.21954

**Published:** 2023-04-01

**Authors:** Serra Örsten, Engin Yılmaz, Yakut Akyön

**Affiliations:** 1 Hacettepe University, Vocational School of Health Services, Ankara, Turkey; 2 Department of Medical Biology, Hacettepe University Faculty of Medicine, Ankara, Turkey; 3 Department of Medical Microbiology, Hacettepe University Faculty of Medicine, Ankara, Turkey

**Keywords:** Clarithromycin, Helicobacter pylori, microbial sensitivity tests, point mutation, sequence analysis

## Abstract

**Background:**

The purpose of this study was to determine the antimicrobial status of stocked clinical *Helicobacter pylori* isolates by using antibiotic gradient test and subsequently identify the mutations that cause clarithromycin resistance by DNA sequencing. Turkey is a transition zone between Europe and Asia; therefore, we also aimed to show both continents’ mutations in Turkish isolates.

**Methods:**

One hundred forty-seven *H. pylori* isolates that had been stocked at −80°C between 1998 and 2008 were randomly selected and included in the study. Antibiotic susceptibility tests were performed using antibiotic gradient test for clarithromycin, amoxicillin, tetracycline, metronidazole, and levofloxacin. A polymerase chain reaction targeting the region of 23S rRNA gene domain V of *H. pylori* was performed and the mutations responsible for resistance against clarithromycin were defined by sequencing.

**Results:**

All of the tested isolates were found susceptible to amoxicillin and tetracycline. However, clarithromycin, metronidazole, and levofloxacin resistance were detected in 28.5% (42/147), 44.8% (66/147), and 23.1% (34/147) of the isolates, respectively. Point mutations were detected in 46 isolates (46/147, 31.2%). The majority of mutations were defined as A2143G (19/46, 41.3%), A2142G (14/46, 30.4%), and A2142C (7/46, 15.2%), respectively. T2188C, T2182C, G1949A, G1940A, and C1944T mutations were also identified in the isolates.

**Conclusion:**

In conclusion, the most common mutations associated with clarithromycin resistance in H. pylori have been identified as A2143G, A2142G, and A2142C which are the most frequently detected mutations in European countries. Same mutations and other mutations like T2182C have also been detected frequently in north-eastern countries and China. Since Turkey is a transition zone between Europe and Asia, Turkey might have strains that carry mutations found in both continents.

**Main Points**

- The aim of this study is to identify the point mutations associated with clarithromycin resistance in *Helicobacter pylori* isolates.
- The observed mutations in this study were previously detected in north-eastern countries and Europe.
- Less frequently observed mutations in *H. pylori *should not be underestimated.

## Introduction

*Helicobacter pylori* (HP) is a Gram-negative bacterium that is the most important causative agent of gastritis, peptic ulcer, gastric cancer, and mucosa-associated lymphoid tissue (MALT) lymphoma.^1,2^ *H. pylori* infection is frequently reported worldwide, and many studies have shown a link between low socioeconomic level and risk of HP infection.^3,4^ In industrialized countries, HP infection prevalence rate has been recorded as 11%, whereas in unindustrialized countries, it has been reported as 83.4%.^5^ Although individual studies show HP infection is common in Turkey, the frequency of HP throughout the country is not known exactly. In a comprehensive study, the weighted overall prevalence was found as 82.5% (95% CI: 81.0-84.2). The prevalence was also found higher in the male gender. Additionally, the region of residence was found to be related to the prevalence of HP infection. The prevalence of HP infection was found lowest in southern (78.7%) and highest in eastern (88.1%) Turkey.^6^ According to previous studies, treatment should always be applied to eradicate HP infection. Eradication of HP is a problem due to antibiotic resistance and patient’s noncompliance. In order to achieve treatment, susceptibility testing should be performed and also the patient should be informed properly.^7^ The standard regime for HP therapy is a combination of a proton pump inhibitor (PPI), or ranitidine bismuth citrate, and 2 antibiotics among amoxicillin, clarithromycin, or metronidazole for 7 days.^8-10^ Increased antimicrobial resistance to metronidazole, levofloxacin, and particularly clarithromycin is the major reason for failure in the eradication of HP infection. In first-line empirical therapy, bismuth-containing quadruple therapy (BCQT) is recommended, while concomitant therapy (CT) can also be chosen in areas where bismuth is not available or where clarithromycin resistance exists.^1^ Although alternative first-line treatments are suggested such as sequential therapy (ST) and hybrid therapy (HT), in the guidelines developed by study groups for the treatment of HP infection, there is no consensus on the best first-line therapy regime.^1^ Therefore, management of HP infection according to drug sensitivity tests provides not only a successful treatment rate but also prevents the overuse of antibiotics.^11,12^

Clarithromycin is a macrolide antibiotic that acts by binding to the 23S rRNA of bacterial ribosomes to inhibit peptide translation. The major reason of clarithromycin resistance in *H. pylori* is point mutations in domain V of the 23S rRNA gene in the 50S ribosomal subunit.^13^ Although the most prevalent mutations are adenine to guanine (A-G) transitions at 2142 and 2143 positions, there are a lot of defined mutations in clarithromycin-resistant strains (A2515G, T2717C, A2116G, G2141A, A2144T, T2182C, G2224A, C2245T, and T2289C).^8,14-16^ Clarithromycin resistance in *H. pylori* has been increasing worldwide, particularly in developing countries including Turkey. The clarithromycin resistance rates have been reported that range between 41%, 28.5%, and 18.2% in northwestern, central, and southern Turkey, respectively.^17^ Therefore, the determination of resistance status in *H. pylori* isolates will be beneficial for treatment management in local health settings.

The aim of this study was to determine the antimicrobial susceptibility status of stocked clinical *H. pylori* isolates by antibiotic gradient test (E-test, BioMérieux/France) and subsequently identify the mutations that cause clarithromycin resistance to show that mutations in isolates by DNA sequencing from Turkey.

## Materials and Methods

### Sample Collection

One hundred forty-seven *H. pylori* isolates which had been stocked at −80°C between 1998 and 2008 were randomly selected and included in the study. As the study involved the evaluation of bacterial strains exclusively and no identifying information of the infected individuals was disclosed, an institutional/regional ethics approval was not necessary. The stocked isolates were retrieved from gastric biopsy samples which were obtained via esophagogastroduodenoscopy. Briefly, stock cultures were subcultured on brain-heart-infusion (BHI) agar containing 7% horse blood, vancomycin (10 mg/L), trimethoprim (5 mg/L), cefzulodine (5 mg/L), and amphotericin B (5 mg/L) (Oxoid SR147 E). Plates were incubated for 10 days at 35-37°C under microaerophilic conditions (8%-10% CO_2_, 5%-6% O_2_, 80%-85% NO_2_, 98% humidity). The confirmation of the stock isolates of *H. pylori* was performed via Gram staining, urease, catalase, and oxidase tests.

### Antibiotic Susceptibility Tests

Subcultures of *H. pylori* were obtained on antibiotic-free BHI agars under microaerophilic conditions for 72 hours. Antibiotic susceptibility tests were performed on Mueller–Hinton agar (MHA) supplemented with 5% horse blood. According to the manufacturer’s instructions, bacterial suspension was prepared as McFarland 3.0 in BHI broth. The clarithromycin, amoxicillin, tetracycline, metronidazole, and levofloxacin gradient strips (E-test Bio Mérieux, France) were placed on every inoculated MHA and were incubated for 3 days under microaerophilic conditions. The minimum inhibitor concentration (MIC) was evaluated using the European Committee on Antimicrobial Susceptibility Testing (EUCAST) criteria. According to EUCAST guidelines, clarithromycin MIC ≥ 1 µg/mL, metronidazole MIC ≥ 8 µg/ml, amoxicillin MIC ≥ 0.5 µg/mL, levofloxacin MIC ≥ 1 µg/mL, and tetracycline MIC ≥ 1 µg/mL was accepted as resistant.

### DNA Extraction, Amplification, and Sequencing

The DNA extraction was performed directly on the stocked cultures by a modified cetyltrimethylammonium bromide method according to the DNA Miniprep protocol of Wilson.^18^ Until sequencing, the obtained DNA was stored at −20°C. The highly conserved region of the 23S rRNA gene domain V of *H. pylori* (GenBank: U27270) was amplified using newly designed forward primer Hp23.1 F 5’ GGC TCT TTG AGT CCT TTT AGG AC 3’ and reverse primer Hp23.1 R 5’ AGC TAA CAG AAA CAT CAA GGG TGG T 3’. The polymerase chain reaction (PCR) temperature cycling conditions were as follows: 94°C for 3 minutes; 35 cycles of denaturation at 94°C for 30 seconds, annealing at 56°C for 30 seconds, and elongation at 72°C for 30 seconds. The final cycle was followed by an extension at 72°C for 3 minutes. The sequencing reaction was performed with BigDye Terminator sequencing kit 3.1 (PE Applied Biosystems, Calif, USA) in line with the manufacturer’s protocol. Reaction products were analyzed with Applied Biosystems 3730XL Genetic Analyzer. To identify conserved regions, an alignment of different *H. pylori* sequences that were available on GenBank was performed using MegAlign software (DNAstar, Madison, Wis, USA). The crude findings were compared to the GenBank and the mutations responsible for resistance against clarithromycin were evaluated. NCTC *H. pylori* 11637 was used as the negative control.

### Statistical Analysis

IBM Statistical Package for Social Sciences Statistics Ver. 20 (IBM Corp.; Armonk, NY, USA) was used for statistical analysis. Categorical data were evaluated with the *χ* ^2^ test.

## Results

A total of 147 isolates were tested against the following antibiotics: clarithromycin, amoxicillin, tetracycline, metronidazole, and levofloxacin. As a result, 42 of the 147 isolates (28.5%) were evaluated as clarithromycin resistant (>0.5 mg/L). All of the tested isolates were found to be susceptible to amoxicillin and tetracycline according to EUCAST clinical breakpoints. However, clarithromycin, metronidazole, and levofloxacin resistance were detected in 28.5% (42/147), 44.8% (66/147), and 23.1% (34/147) of the isolates, respectively. For all tested antibiotics, the MIC50, MIC90, and MIC range data were given in Table 1. Some of the isolates were found to be resistant to several antibiotics, simultaneously. In detail, 11 isolates were resistant to clarithromycin, metronidazole, and levofloxacin; 13 isolates were identified as clarithromycin and metronidazole resistant. Besides, 8 isolates were resistant to metronidazole and levofloxacin, 2 isolates were found to be resistant to clarithromycin and levofloxacin.

Out of 147 isolates, 101 (68.7%) of them did not harbor mutations that would be responsible of clarithromycin resistance. Point mutations were detected in 46 of the isolates (46/147, 31.2%). In 19 (19/46, 41.3%) A2143G, in 14 (14/46, 30.4%) A2142G, and in 1 of these isolates (2.1%), A1983G variation was detected; in 7 (7/46, 15.2%) A2142C, in 2 (4.3%) T2188C, in 2 (4.3%) T2182C, in 2 G1949A (2%), and in 1 of them, G1940A and C1944T (2%) mutations were detected. Clarithromycin resistance was found statistically significant in *H. pylori *strains with the point mutations (*P *< .05). However, certain point mutations (T2188C, G1949A, G1940A, and C1944T) were not associated with clarithromycin resistance. The antimicrobial sensitivity findings of the isolates are presented in Table 2. The mutation types of multiresistant strains are given in Table 3.

## Discussion

According to the expert recommendations, the treatment for HP infection should be successful at the first implementation.^19,20^ Proton pump inhibitor triple therapy which is the use of 2 antibiotics accompanying the PPI is still effective in regions with low drug resistance. However, the success rate of PPI triple therapy with clarithromycin-amoxicillin has decreased from 90% to less than 70%. The main factor in this decline is the increasing resistance to clarithromycin which is the most effective antibiotic in the treatment of HP infections.^21^ According to Maastricht V/Florence Consensus Report, triple therapy containing metronidazole or BCQT should be applied in regions where the clarithromycin resistance displays higher than 15%.^20^ The reported clarithromycin resistance rates vary worldwide. As an example, reported rates from Italy and Japan are ∼30%, from Turkey ∼40%, from China ∼50%, and from Sweden and Taiwan ∼15%.^8,17^ In the present study, the clarithromycin resistance rate was found to be 28.5% (42/147) via the E-test method. The antimicrobial resistance of *H. pylori* is mainly determined using E-test and agar dilution methods in Turkey.^22,23^ In this study, the clarithromycin rate (28.5%) has been found higher than the proposed threshold of 15%, but it is consistent with the determined overall resistance of *H. pylori* to clarithromycin between 1999 and 2015 (24.8%) in Turkey.^24^ The clarithromycin and metronidazole resistance rates from different regions of Turkey were reported by many research groups. According to these reports, clarithromycin and metronidazole resistance rates were found to be 28.5% and 39.2% in central Anatolia, 18.2% and 45.5% in Southern Anatolia, respectively, in 2012. From Northwestern Anatolia, corresponding rates of resistance were reported as 36.7% and 35.5% in 2014 and 41.9% in 2009. In addition, the reported clarithromycin resistance rate in Southern Anatolia was 8.8% in 2013.^17,22,25-27^ In a recent study from the Eastern Black Sea region of Turkey, the clarithromycin resistance rate was reported as 28.2%.^28^ Considering all these studies, clarithromycin resistance in Turkey has shown a tendency to increase over the years.

The failure of *H. pylori* eradication is influenced by clarithromycin resistance associated with a 23S rRNA point mutation.^29^ Hence, the identification of point mutations using molecular methods is valuable for the management of therapy. Aforementioned, A2143G, A2142G, and A2142C are the most frequently detected point mutations in the region of 23S rRNA and are responsible for the majority of clarithromycin-resistant cases.^8,29,30^ The A2143G mutation has a great impact on the development of clarithromycin resistance especially in European countries with rates of 85.0% and 90.0% in Spain and France, respectively.^31-33^ However, data on clinical significance of other mutations detected in *H. pylori* isolates are limited.^29^ The main aim of this study was to identify the point mutations which are responsible for the clarithromycin resistance. In the present study, A2143G (19/46, 41.3%), A2142G (14/46, 30.4%), and A2142C (7/46, 15.2%) mutations were found in most of the isolates and all of them were determined as clarithromycin resistant. These mutations are most frequently reported in *H. pylori* isolates from Turkey, and these findings are consistent with previous studies.^8^ In addition, T2182C mutation was detected in 2 isolates (2/46, 4.3%) determined as clarithromycin resistant. However, there have been conflicting results on association of clarithromycin resistance regarding T2182C mutation. This mutation is mainly associated with clarithromycin resistance in Korea; however, its clinical importance has not been enlightened yet.^29,30,34,35^ Therefore, comprehensive studies are needed to determine the clinical significance of T2182C mutation.

On the other hand, several mutations were identified in clarithromycin-susceptible isolates and should not be neglected. In 2 isolates T2188C (2/46, 4.3%), mutation was detected. One of these 2 also had G1949A (1/46, 2%) and the other had both G1940A and C1944T (1/46, 2%) mutations. In a recent report from Iran, researchers suggest that T2188C is considered as being among the putative point mutations involved in clarithromycin resistance.^36^ In contrast, T2188C mutation has been detected in clarithromycin-susceptible isolates in the present study. Similarly, G1949A has been identified in clarithromycin-susceptible isolates. However, according to a previous study, G1949A may not be only related to clarithromycin resistance but may also synergistically promote resistance at A2143G.^37^ Therefore, even if resistance development is not yet observed, mutations should be identified by sequence analysis and less frequently observed mutations should not be underestimated.

A limitation of this study is that the sample group included only stocked *H. pylori* isolates. Thus, further prospective studies are needed to show that these mutations do cause clarithromycin resistance in *H. pylori*.

In conclusion, the most common mutations associated with clarithromycin resistance in *H. pylori* are A2143G, A2142G, and A2142C mutations, and they are most frequently detected in European countries. Other common mutations are those detected in north-eastern countries and China. Since Turkey is a transition zone between Europe and Asia, Turkey might have mutations found in both continents. In this study, although other mutations were detected in the sequenced region of the 23S rRNA gene domain V of *H. pylori*, further prospective studies are needed to show that these mutations do cause clarithromycin resistance in *H. pylori*.

## Figures and Tables

**Table 1.** Antimicrobial MIC Values of *Helicobacter pylori* Isolates

Antibiotics	MIC50 (µg/mL)	MIC90 (µg/mL)	MIC Range (µg/mL)
Clarithromycin	0.25	128	0.016-256
Levofloxacin	0.064	6	0.002-256
Metronidazole	4	256	0.016-256
Amoxicillin	0.016	0.032	0.016-0.032
Tetracycline	0.016	0.032	0.016-0.032

MIC, minimum inhibitor concentration.

**Table 2.** The Antimicrobial Sensitivity Findings of the Isolates

Antimicrobial Susceptibility	Number of Isolates (n = 147)				
	Clarithromycin	Levofloxacin	Metronidazole	Amoxicillin	Tetracycline
Resistant	42 (28.5%)	34 (23.1%)	66 (44.8%)	0 (0%)	0 (0%)
Susceptible	105 (71.5%)	113 (76.9%)	81 (55.2%)	147 (100%)	147 (100%)

**Table 3.** Multiple Resistant Strains with Point Mutation

Point Mutations	Isolate Number	Clarithromycin	Levofloxacin	Metronidazole
A2143G	1	R	R	R
	2	R	R	R
	3	R	R	R
	4	R	S	R
	5	R	S	R
	6	R	S	R
	7	R	S	R
	8	R	S	R
	9	R	S	R
	10	R	R	S
	11	R	S	R
	12	R	R	S
A2142G	13	R	R	R
	14	R	R	R
	15	R	S	R
	16	R	S	R
	17	R	S	R
	18	R	S	R
	19	R	S	R
A2142G, A1983G	20	R	R	R
A2142C	21	R	R	R
	22	R	R	R
	23	R	R	R
	24	R	R	R
	25	R	S	R
T2182C	26	R	R	R

S, susceptible; R, resistant.
